# mHealth intervention delivered in general practice to increase physical activity and reduce sedentary behaviour of patients with prediabetes and type 2 diabetes (ENERGISED): rationale and study protocol for a pragmatic randomised controlled trial

**DOI:** 10.1186/s12889-023-15513-1

**Published:** 2023-03-31

**Authors:** Tomas Vetrovsky, Norbert Kral, Marketa Pfeiferova, Jitka Kuhnova, Jan Novak, Charlotte Wahlich, Andrea Jaklova, Katerina Jurkova, Michael Janek, Dan Omcirk, Vaclav Capek, Iris Maes, Michal Steffl, Michael Ussher, James J. Tufano, Steriani Elavsky, Delfien Van Dyck, Richard Cimler, Tom Yates, Tess Harris, Bohumil Seifert

**Affiliations:** 1 Faculty of Physical Education and Sport, Charles University, Prague, Czech Republic; 2 Institute of General Practice, 1st Faculty of Medicine, Charles University, Prague, Czech Republic; 3 Faculty of Science, University of Hradec Kralove, Hradec Kralove, Czech Republic; 4 Population Health Research Institute, St George’s University of London, London, UK; 5 2nd Faculty of Medicine, Charles University, Prague, Czech Republic; 6 Department of Movement and Sports Sciences, Ghent University, Ghent, Belgium; 7 Institute for Social Marketing and Health, University of Stirling, Stirling, UK; 8 Department of Human Movement Studies, University of Ostrava, Ostrava, Czech Republic; 9 Diabetes Research Centre, University of Leicester, Leicester, UK; 10 National Institute for Health Research (NIHR) Leicester Biomedical Research Centre, University Hospitals of Leicester NHS Trust and the University of Leicester, Leicester, UK

**Keywords:** Step-count, Just-in-time adaptive intervention (JITAI), Primary care, Fitbit, Active control, Self-monitoring, Ecological Momentary Assessment (EMA), Micro-randomisation, Phone counselling, Text messages

## Abstract

**Background:**

The growing number of patients with type 2 diabetes and prediabetes is a major public health concern. Physical activity is a cornerstone of diabetes management and may prevent its onset in prediabetes patients. Despite this, many patients with (pre)diabetes remain physically inactive. Primary care physicians are well-situated to deliver interventions to increase their patients' physical activity levels. However, effective and sustainable physical activity interventions for (pre)diabetes patients that can be translated into routine primary care are lacking.

**Methods:**

We describe the rationale and protocol for a 12-month pragmatic, multicentre, randomised, controlled trial assessing the effectiveness of an mHealth intervention delivered in general practice to increase physical activity and reduce sedentary behaviour of patients with prediabetes and type 2 diabetes (ENERGISED). Twenty-one general practices will recruit 340 patients with (pre)diabetes during routine health check-ups. Patients allocated to the active control arm will receive a Fitbit activity tracker to self-monitor their daily steps and try to achieve the recommended step goal. Patients allocated to the intervention arm will additionally receive the mHealth intervention, including the delivery of several text messages per week, with some of them delivered just in time, based on data continuously collected by the Fitbit tracker. The trial consists of two phases, each lasting six months: the lead-in phase, when the mHealth intervention will be supported with human phone counselling, and the maintenance phase, when the intervention will be fully automated. The primary outcome, average ambulatory activity (steps/day) measured by a wrist-worn accelerometer, will be assessed at the end of the maintenance phase at 12 months.

**Discussion:**

The trial has several strengths, such as the choice of active control to isolate the net effect of the intervention beyond simple self-monitoring with an activity tracker, broad eligibility criteria allowing for the inclusion of patients without a smartphone, procedures to minimise selection bias, and involvement of a relatively large number of general practices. These design choices contribute to the trial’s pragmatic character and ensure that the intervention, if effective, can be translated into routine primary care practice, allowing important public health benefits.

**Trial registration:**

ClinicalTrials.gov (NCT05351359, 28/04/2022).

**Supplementary Information:**

The online version contains supplementary material available at 10.1186/s12889-023-15513-1.

## Background

The growing number of patients with type 2 diabetes is a major worldwide public health concern [1]. Functioning as both a treatment and countermeasure, physical activity (PA) is a cornerstone of diabetes management and may prevent or delay its onset in patients with prediabetes [2, 3]. Specifically, moderate to high volumes of aerobic activity are associated with substantially lower cardiovascular and overall mortality risks in patients with type 2 diabetes [4]. Furthermore, regular PA can also reduce glycated haemoglobin (HbA_1c_), triglycerides, blood pressure, and insulin resistance in diabetes patients [5–7]. Similarly, in individuals with prediabetes, PA interventions have shown a favourable effect on oral glucose tolerance, fasting blood sugar, and HbA_1c_ levels [8, 9]. As a result, current guidelines recommend that adults with (pre)diabetes should engage in at least 150 to 300 min or more of at least moderate-intensity aerobic activity, which should be spread throughout the week, and break up sitting with small doses of PA throughout the day [10, 11].

Despite the proven benefits and clear recommendations, many patients with (pre)diabetes remain physically inactive [12, 13]. Since most (pre)diabetes patients regularly visit their primary care physicians, these are well-situated to play a significant role in delivering interventions to increase their patients' PA levels [14, 15]. Despite this potential, delivering evidence-based PA interventions for (pre)diabetes patients in primary care settings is not common practice [16]. One possible explanation is that many interventions are designed to achieve maximum effect in explanatory trials but are not fully integrated into primary care and thus are difficult to translate into routine practice [17]. Indeed, some of the interventions were shown to be effective in early-stage trials but failed when moved along the translation continuum. For example, a pedometer-based, very brief intervention showed promise in a preliminary trial [18]. When scaled up to routine delivery in the context of preventive health checks in 23 primary care practices, it showed no benefit due to suboptimal fidelity of delivery [19]. Furthermore, the Walking Away educational program for prediabetes patients demonstrated good efficacy [20] in a small-scale trial and was commissioned into routine primary care. However, a subsequent cluster randomised trial involving 10 general practices showed only a modest increase in walking activity of around 400 steps at 12 months, and this effect completely disappeared at 36 months [21]. Thus, effective and sustainable PA interventions for (pre)diabetes patients that can be translated into routine primary care are lacking and urgently needed [22].

### Rationale for the intervention

Primary care physicians, such as general practitioners (GPs), are commonly viewed as credible sources of health information, and they themselves believe that they should play a role in promoting PA among their patients [23–25]. However, well-recognised barriers make implementing PA counselling in primary care difficult, with a lack of time due to conflicting priorities being one of the most critical barriers [26–28]. Thus, an ideal intervention must be smoothly integrated within primary care routines and only require a minimum amount of time and effort from the GPs [28]. Nevertheless, the intervention should still benefit from GPs' personal involvement in the form of brief advice to ensure that the intervention is credible and taken seriously by the patients [29, 30]. Although a one-off piece of PA advice is commonly provided, it only has a limited impact on a patient's PA levels [31]. To make the advice more effective, a specific goal can be provided [32, 33], and a simple activity tracker can be used to help the patient self-monitor their progress towards that goal [34–36].

Nevertheless, multiple contacts, including one with a primary care physician, are needed to achieve a significant clinical effect. Specifically, PA interventions with at least five contacts have a larger effect than those with fewer contacts [37]. To ensure patient convenience, these contacts can be made remotely, for example, in the form of phone counselling which has been recently demonstrated as a particularly effective component of PA interventions [38]. Though beneficial, increasing the number of contacts requires substantial resources, and even then, the effect is difficult to maintain in the long term [39, 40]. For example, in the PACE-UP trial of 1023 primary care patients 45—75 y old, three practice nurse consultations and a pedometer increased the daily step count by a modest 500 steps compared to the pedometer alone after three months. However, after 12 months, the difference disappeared [41].

Considering this, complementing GP's brief advice with the prescription of digital therapeutics based on mobile health (mHealth) technologies can potentially increase the long-term effectiveness of PA interventions [42–45]. While the development of mHealth interventions carries a substantial cost, once deployed, they are relatively cheap to maintain and adapt. Thus, they can be sustained long-term and scaled up for translation to standard practice. For example, in the PROPELS trial of 1366 prediabetes patients, a combination of a group-based education programme and mHealth follow-on support increased ambulatory activity of prediabetes patients by 550 steps/day at 12 months when compared to usual care control [46].

Most mHealth interventions to date delivered prompts (app notifications or text messages) at fixed or random times [47]. However, providing prompts when someone is not receptive can result in frustration and disengagement with the intervention [48–50]. These limitations can be overcome by just-in-time adaptive interventions (JITAI), which use data from wearable sensors to intervene when it is most relevant for the patient. For example, they can prompt patients to interrupt sitting when they are actually sitting for a prolonged time or to walk faster when they are actually walking [51–53].

In conclusion, an effective and sustainable PA intervention for (pre)diabetes patients with the potential for translation to routine primary care should harness novel mHealth technologies based on JITAI principles but still involve personal delivery of brief PA advice by GPs, including the provision of a specific goal and an activity tracker for self-monitoring. To boost GPs' one-off piece of advice and facilitate the adoption and tailoring of the mHealth component, the intervention should also employ a limited number of phone counselling sessions during its lead-in phase.

### Objective

In this paper, we describe the protocol of a randomised controlled trial assessing the effectiveness of the mHealth intervention delivered in general practice to increase PA and reduce sedentary behaviour of patients with prediabetes and type 2 diabetes (ENERGISED). The mHealth intervention includes the delivery of several text messages per week, with some of them delivered just in time, based on the data continuously collected by sensors embedded in a Fitbit activity tracker worn by patients. The intervention is introduced to the patients by their GPs during a routine health check-up and is initially supported by monthly phone counselling sessions delivered by trained counsellors. The trial has been designed as a pragmatic trial to ensure that the intervention, if shown to be effective, can be translated into routine practice in primary care settings [17, 54].

## Methods

### Trial design

**Fig. 1:**
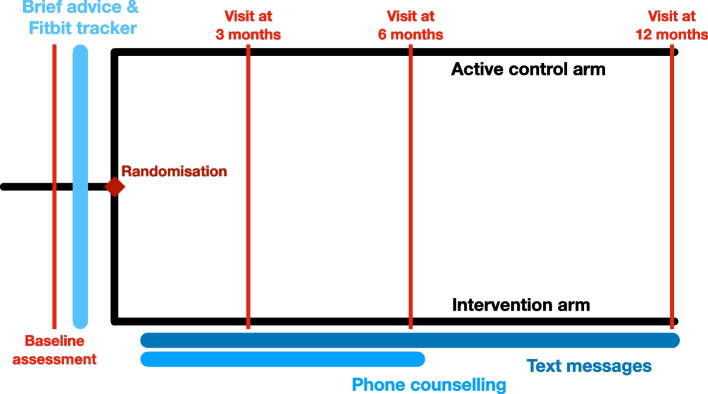
Trial scheme

This 12-month pragmatic, multicentre, randomised, controlled trial with two parallel arms consists of two phases, each lasting for six months: the lead-in phase, when the mHealth intervention is supported with human phone counselling, and the maintenance phase, when the mHealth intervention is fully automated, without any human support. The primary outcome, average ambulatory activity (steps/day) measured by a wrist-worn accelerometer Actigraph, will be assessed at the end of the maintenance phase at 12 months. A trial scheme is shown in Fig. 1.

Patient recruitment started in April 2022 and is expected to be completed in December 2023. This paper is written in accordance with the Standard Protocol Items: Recommendations for Interventional Trials (SPIRIT) 2013 Statement (Additional file 1) and the SPIRIT flow diagram is depicted in Table 1 [55]. The intervention description follows the Template for Intervention Description and Replication (TIDieR) guide (Additional file 2) [56]. Details about the pragmatic design of the ENERGISED trial are provided in the PRagmatic Explanatory Continuum Indicator Summary (PRECIS-2) table of scores for trial domains and the PRECIS-2 wheel scheme (Additional file 3) [57]. The trial is registered in ClinicalTrials.gov (identifier: NCT05351359, registered 28/04/2022, https://clinicaltrials.gov/ct2/show/NCT05351359).

**Table 1:**
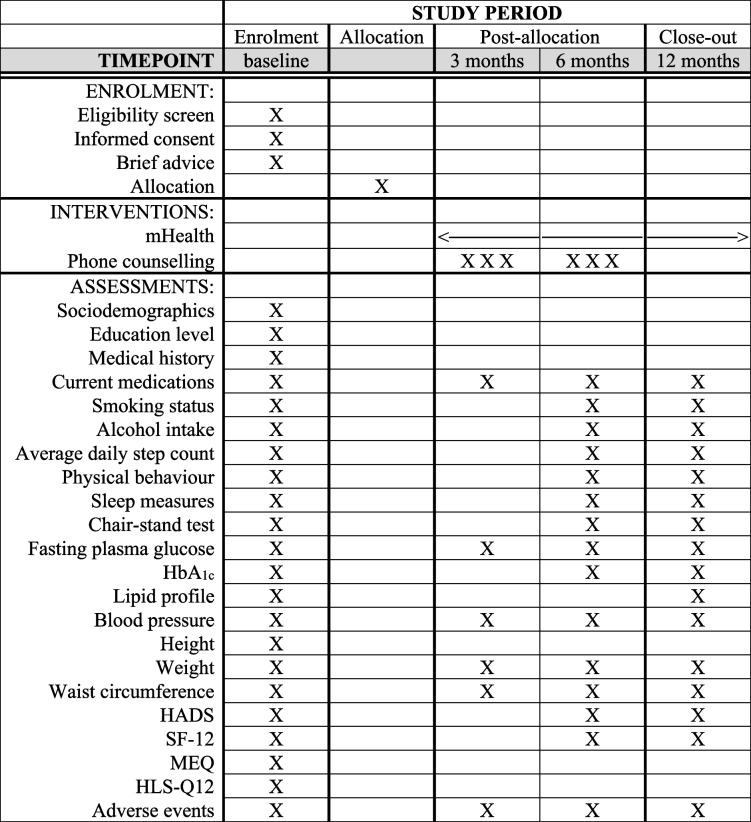
SPIRIT flow diagram

*HbA*_*1c*_ glycated haemoglobin, *HADS* Hospital Anxiety and Depression Scale, *SF-12* 12-Item Short Form Health Survey, *MEQ* Morningness-Eveningness Questionnaire, *HLS-Q12* Short version of the European Health Literacy Survey

### General practice recruitment and training

A convenience sample of 21 general practices has been recruited to participate in the study, representing various urban (n = 11) and rural (n = 10) locations and geographic regions (10 out of 14 regions) across the Czech Republic. Practices were recruited by dissemination at a local conference, in a medical journal for GPs, through emailed invitations, and word-of-mouth.

The participating GPs were trained remotely using Zoom in three separate sessions (each lasting approximately one hour) covering various aspects of the trial conduct, such as using the electronic data capture tool and initialising and downloading data from Actigraphs. Special focus was placed on training the GPs to provide brief PA advice based on the methods specifically developed for PA counselling in primary care [41, 58]. To understand the challenges for their patients, GPs received their own Fitbit activity tracker prior to starting patient recruitment and were asked to self-monitor their PA levels for at least one week. To compensate for their time spent with the trial procedures, the GPs receive remuneration of approximately 100 EUR per patient completing the study.

### Patient eligibility

To be eligible for the trial, patients must meet the following inclusion criteria at randomisation: (1) Diagnosis of prediabetes or type 2 diabetes according to the Czech guidelines for GPs [59, 60], i.e. fasting plasma glucose 5.6–6.9 mmol/l, or 2-h plasma glucose of 7.8–11.0 mmol/l after ingestion of 75 g of oral glucose load for the diagnosis of prediabetes, and fasting plasma glucose ≥ 7.0 mmol/l, or 2-h plasma glucose ≥ 11.1 mmol/l after ingestion of 75 g of the oral glucose load for the diagnosis of type 2 diabetes. (2) Age 18 years or older. (3) Followed for prediabetes/diabetes by a participating GP. Of note, in the Czech Republic, only uncomplicated type 2 diabetes patients with glycated haemoglobin HbA_1c_ ≤ 53 mmol/mol and not taking insulin are commonly followed by a GP; other type 2 diabetes patients are usually followed by a specialist-diabetologist. (4) Regular users of a mobile phone (not necessarily a smartphone), able and willing to answer calls and read text messages as part of the study. (5) Able and willing to wear and use a wrist-worn Fitbit activity tracker for the study duration. (6) Written informed consent provided before any assessment related to the study.

Patients are excluded from the trial if they are: (1) Unable to walk for any reason. (2) Pregnant. (3) Having a household member already recruited for this study to avoid contamination. (4) Living in a residential or nursing care home where the imposed regime could interfere with the intervention. (5) Having any co-morbid conditions that would seriously affect their adherence to the trial procedures (e.g., active malignancy; recent (< 3 months) myocardial infarction, coronary artery bypass graft or cerebrovascular accident; renal disease requiring dialysis; neurological condition (e.g., Parkinson disease); cognitive impairment, or significant hearing or visual impairment; hip or knee joint replacement within three months; major surgery planned within the next 12 months).

### Sample size

To detect a difference of 1000 steps/day at 12 months between arms, with a power of 80%, using a 2-sided 0.05 significance level (alpha), and anticipating the standard deviation of 3000 steps/day [21, 40, 46], 143 subjects per arm (286 in total) will be needed. To account for an expected attrition rate of approximately 15% [21, 40, 46], we plan to recruit 340 patients for the trial. The increase of 1000 steps/day is equivalent to about 10 min/day of brisk walking [61] and equates to around a 4% decrease in the risk of cardiovascular morbidity and mortality in individuals at high cardiovascular risk with impaired glucose tolerance [62].

### Patient recruitment and consent

To achieve the target of 340 recruited patients, each of the 21 general practices has been asked to recruit at least 17 patients. To compensate for potential under-recruitment at individual practices, we will allow those practices that reach this target to continue recruiting until 24 patients.

Previous studies of PA interventions in primary care identified a selection bias when GPs preferentially pick those patients whom they believed to be able to use (e.g., highly educated patients) and to benefit from the intervention (e.g., patients with overweight and obesity) [29, 63]. To minimise the selection bias, we first compiled a list of all prediabetes and type 2 diabetes patients for each general practice based on their computerised medical records. At the start of the recruitment, each general practice was provided with a random selection of 24 patients from their list, i.e., 504 patients in total for all 21 practices, stratified by sex (female:male in 1:1 ratio) and condition (prediabetes:diabetes in 1:2 ratio). GPs were instructed to evaluate the eligibility criteria and introduce the study opportunistically to all eligible patients from this selection whenever they appear in the general practice for a routine health check-up [64]. For patients who are excluded or refuse to participate, GPs record the reasons. When a GP expends all the patients from this selection, they will be offered a new random selection of 12 patients from their original list. This procedure can be repeated until the respective practice exhausts its original list or reaches 24 recruited patients.

When introducing the study, GPs will mention that all enrolled patients receive a Fitbit activity tracker that they can retain after completion of the study, irrespective of the trial arm allocation. Those who agree to participate will be asked to sign a written informed consent, including consent for later access to GP records to assess long-term health outcomes. The patients will also consent for the researchers to access their PA data on their Fitbit account in line with the legal requirements.

To minimise the effect of seasonal variability on PA findings, we will attempt to spread patient recruitment evenly across 12 consecutive months. For example, GPs will be only provided with two Actigraph devices necessary for a week-long baseline assessment, so a GP cannot recruit more than four patients per month.

### Procedures common for all recruited patients

#### First baseline visit to the GP: Baseline assessment

After receiving the patient's consent, a GP will complete a baseline assessment, fit the patients with an accelerometer Actigraph wGT3X-BT (Actigraph, Pensacola, FL, USA) and instruct them to wear it on a non-dominant wrist for seven consecutive days, 24 h a day, while maintaining their typical PA and then to come back for the second visit to return the device.

#### Second baseline visit to the GP: Brief advice and Fitbit tracker

At the second visit, after returning the Actigraph, a GP will provide all patients with a wearable activity tracker Fitbit Inspire 2 (Fitbit Inc., San Francisco, CA) and instruct them to wear it for the duration of the study [65, 66]. The Fitbit tracker requires an initialisation to measure and display the step count. Thus, a GP will ask the patients to initialise the Fitbit tracker by connecting it via Bluetooth to the Fitbit app on their smartphones, using a Fitbit account pre-set by the research team (account login and password will be included in the Fitbit packaging). Patients without their own smartphones will be recommended to ask their relatives or friends who own a smartphone to perform the initialisation for them. Patients who already use their own activity tracker are allowed to wear it instead of the provided Fitbit Inspire 2. However, for the mHealth intervention to function optimally, their tracker must be a Fitbit model equipped with a heart rate sensor, and the patients must link it with the Fitbit account provided by the research team. The patients will be then instructed to maintain their typical PA for one more week while wearing the Fitbit to set their baseline steps.

All patients will also receive from their GPs brief PA advice, an educational leaflet on PA and exercise, and a receipt with a prescription of specific PA goals. Specifically, the GPs will recommend the patients to self-monitor their daily step count and increase it by at least 3000 steps over their baseline steps (as determined during the first week of Fitbit wear). The GPs will advise the patients to achieve this goal gradually over at least six weeks and to perform these extra 3000 steps by an intentionally brisk walk. Brisk 3000 steps approximately translate to 30 min of moderate PA a day [61], consistent with the latest PA recommendations [10, 11] and findings that 30 min of moderate-intensity PA at least five days per week substantially reduces the risk of type 2 diabetes [2]. Furthermore, the GPs will recommend the patients to interrupt prolonged sitting with short bouts of walking or exercise every 30 min [67, 68].

#### Randomisation

After the second visit to the general practice, patients will be randomly allocated in a 1:1 ratio to either the intervention or the active control arm. The randomisation will be performed by the principal investigator centrally, using a computer-automated randomisation system within REDCap electronic data capture tools [69] to guarantee adequate allocation concealment. The trial will use a randomisation scheme stratified by prediabetes/type 2 diabetes condition and sex to ensure equal representation in the arms.

#### Blinding

Due to the nature of the study protocol, the patients and researchers cannot be blinded, as they will both be aware of the allocation due to their active role in the intervention. However, the participating GPs, who will conduct all the assessments, will be blinded to arm allocation unless they specifically ask a patient about their allocation (which the GPs are discouraged from doing). All those conducting the data analysis will also be blinded to group allocation.

#### Phone contact by the research team member

The patients will be made aware of their allocation by a research team member via a phone call. During this call, the research team member will also check if the patient has successfully initiated the Fitbit (by asking the patient) and is regularly syncing it with their account (by checking the account directly). Patients who experience trouble with the initialisation or syncing will be provided with basic technical support during the call to ensure that as many patients as possible continuously sync their Fitbit data for analytical purposes.

The research team member will check monthly if the patients continue syncing their Fitbits; if they do not, they will be contacted by phone and reminded to do so. The patients unable to sync their Fitbits despite the support and those without their own smartphone will be asked to self-monitor by keeping a paper-based diary with daily records of their step count as measured by Fitbit.

To prevent interference of push notifications built into the Fitbit app (e.g., reminders to move, notifications of step goal milestones) with our study, all these notifications will be remotely switched off by the research team member. In addition, the research team member will instruct the patients not to change the settings of their Fitbit app.

#### Visits to the GP at 3, 6, and 12 months

During the study period, all patients will visit their GPs for a routine health check-up every three months as recommended by the Czech guidelines for GPs [59, 60]. During visits at 3, 6, and 12 months, they will undergo study assessments (Table 1). Patients who finish the study can retain the Fitbit device.

### Active control arm

As previously stated, the patients from the active control arm will receive from their GPs brief PA advice, an educational leaflet, and a receipt with a PA prescription at baseline. They will also receive a Fitbit tracker to self-monitor their daily steps and try to achieve the recommended goal of an extra 3000 daily steps (Table 2). We chose the active control rather than a passive control to isolate the net effect of the mHealth intervention (see the Discussion section for the elaborate rationale for choosing the active control) [38].

**Table 2:** Intervention components and behaviour change techniques used in the active control and intervention arms

Active control arm	Intervention arm	Component	Behaviour change techniques^a^
✓	✓	Brief physical activity advice from the general practitioner at baseline, including:	9.1 Credible source
✓	✓	- educational leaflet	4.1 Instruction on how to perform the behaviour 1.4 Action planning 5.1 Information about health 8.7 Graded task
✓	✓	- receipt with physical activity prescription and a specific goal	1.1 Goal setting (behaviour)
✓	✓	- provision of the Fitbit activity tracker	2.3 Self-monitoring of behaviour
	✓	Text messages for the entire study duration (12 months):	
	✓	- just-in-time prompts to increase walking pace (triggered when the patient is walking for 5 consecutive minutes)	7.1 Prompts/cues 8.3 Habit formation
	✓	- just-in-time prompts to interrupt sitting (triggered when the patient sits for more than 30 min)	7.1 Prompts/cues 8.2 Behaviour substitution
	✓	- interim review of the patient’s weekly step goal (triggered on Friday evening)	1.5 Review behaviour goal 1.6 Discrepancy between current behaviour and goal
	✓	- feedback on the patient's weekly performance and encouragement for the upcoming week (triggered on Sunday evening)	2.2 Feedback on behaviour 10.4 Social reward
	✓	- reminders of the action plan (adapted to specific plans of each patient)	1.4 Action planning 8.3 Habit formation
	✓	- occasional educational messages	5.1 Information about health consequences
	✓	Phone counselling sessions at 2 weeks, then monthly till the six months (seven sessions in total)	1.1 Goal setting (behaviour) 1.2 Problem solving 1.4 Action planning 1.5 Review behaviour goal 2.2 Feedback on behaviour 3.1 Social support 8.7 Graded task 10.4 Social reward

^a^The Behaviour Change Techniques were coded using the taxonomy by Michie et al. [72]

### Intervention arm

The patients in the intervention arm will be exposed to the same procedures as those from the active control arm. In addition, they will also receive the mHealth intervention incorporating JITAI principles (Table 2). During the first six months, the mHealth intervention will be supported with human phone counselling (lead-in phase). Then, for the next six months, the mHealth intervention will be fully automated, without any human support (maintenance phase) (Fig. 1).

#### mHealth intervention

The mHealth intervention has been developed with the involvement of (pre)diabetes patients according to the mHealth development and evaluation framework [70, 71]. This framework consists of four sequential phases: (a) conceptualisation, (b) formative research based on focus groups with the target population, (c) pre-testing of specific messages, and (d) piloting [71]. The intervention development and the messages' content and triggering rules will be described in a separate paper. In brief, the patients receive six types of text messages involving various behaviour change techniques (Table 2) [72]. (1) Just-in-time prompts to increase walking pace are triggered when the patient is actually walking (5 consecutive minutes with steps per minute in the range from 60 to 100). (2) Just-in-time prompts to interrupt sitting are sent when the patient sits for more than 30 min. (3) Interim reviews of patients’ weekly step goals are triggered on Friday evening, highlighting the potential discrepancy between the goal and actual step count so that the patients have a chance to catch up over the weekend. (4) At the end of the week, on Sunday evening, patients receive feedback on their weekly performance and encouragement for the upcoming week. (5) Reminders of the action plans are adapted to specific plans of each patient as set during the phone counselling session (e.g., patients are reminded to go for a short walk after lunch or to walk their dog on a pre-specified day and time). (6) Finally, short educational messages are sent occasionally to highlight the importance of PA in (pre)diabetes management.

**Fig. 2:**
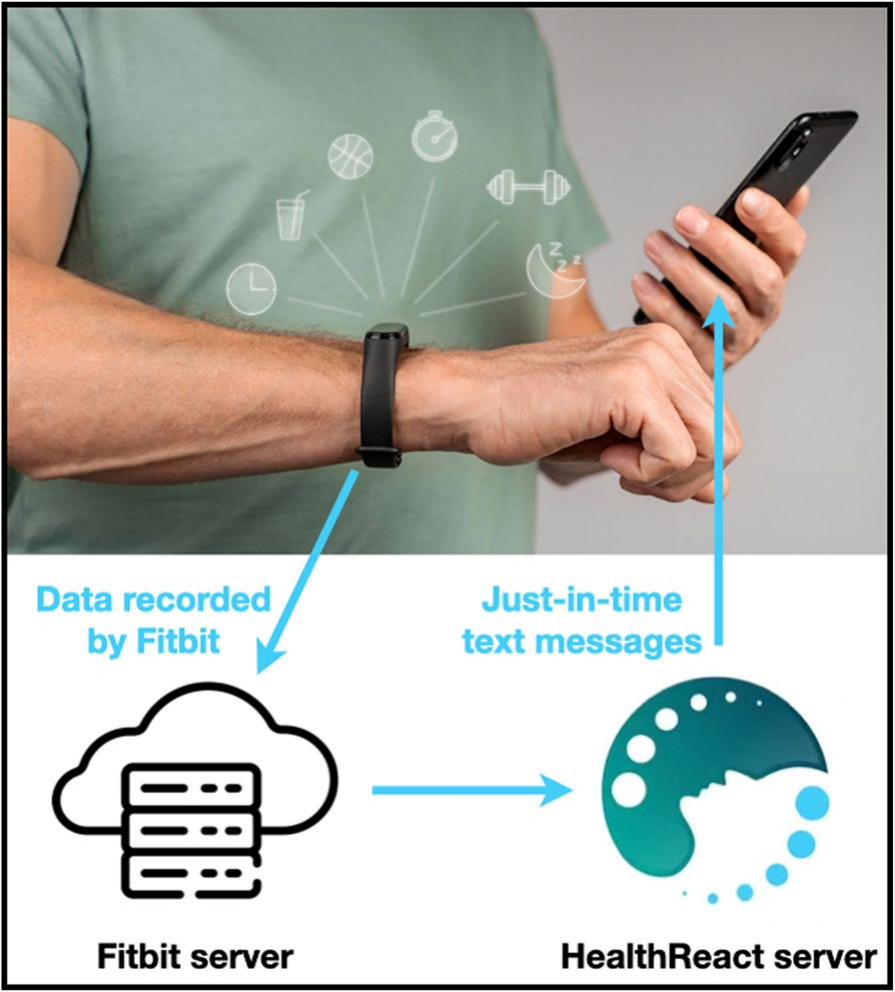
Scheme of the HealthReact system. The HealthReact system triggers sending out just-in-time text messages based on data recorded by the Fitbit tracker

The mHealth intervention is enabled by the HealthReact system developed at the University of Hradec Kralove [73]. HealthReact consists of a server-side application connected to the Fitbit server and thus can trigger sending out just-in-time text messages based on the data recorded by the Fitbit tracker (Fig. 2). Researchers can choose from a wide variety of just-in-time triggers to suit study objectives and adapt the triggers to the specific needs of individual patients. The HealthReact system enables the setting of certain parameters to regulate the number of text messages sent (e.g., total number of just-in-time prompts per day, the minimum time between two prompts, the time window when prompts are triggered, the probability that a triggered text message is actually sent out). These parameters are tuned so that the total number of text messages per week ranges between 4 and 6.

For the mHealth intervention to function optimally, patients must be equipped with a smartphone able to run the Fitbit app (Android 8.0 or iOS 14.0 and later as of January 2023) with a continuous internet connection. Patients who fall short of such requirements will receive adapted mHealth intervention that is equalised for the total number of text messages and the behaviour change techniques used. For example, in patients without a mobile data plan who only sync their mobile when connected to wifi, the prompts to increase their walking pace could not be triggered just in time when they walk, but would still be sent out at a pre-specified fixed time. In patients that do not sync reliably even when at home, the prompts to interrupt sitting could not be triggered just in time and would be replaced with a general reminder to regularly interrupt sitting. Finally, in patients without a smartphone or unable to sync any Fitbit data to HealthReact for other reasons, messages reviewing their step goal and providing feedback could not be personalised, but would still be sent out as simple reminders to review the set goal and provide encouragement.

#### Phone counselling

To facilitate the adoption of the mHealth intervention, counsellors recruited from among the university students and trained by the research team members (CW, TV, JK, JN) will contact the patients by phone at the start of the intervention, at months 1, 2, 3, 4 and 5, and after the six-month assessment (i.e., seven phone calls altogether, each lasting approximately 10 to 20 min). In addition to enabling the implementation and tailoring of the mHealth intervention to individual patients, the counsellors will use various behavioural change techniques (Table 2) to support patients in the desired behavioural change and enhance their adherence to the intervention.

During the first phone call, approximately one to two weeks after the second baseline visit to the GP, the counsellor will check the patients’ baseline mean daily step count as recorded by Fitbit during the previous week when the patients were supposed not to change their physical behaviour. The counsellor will initially recommend the patients increase their daily step count by at least 3000 above their baseline gradually over at least six weeks. However, if patients are not comfortable with the increase of 3000 steps, they can suggest a goal that they feel is more realistic. Importantly, the counsellor will make sure that the patients have a goal that they consider their own and not imposed on them by the counsellor [74, 75]. Finally, the daily step goal will be converted to a weekly step goal (multiplying by 7), allowing more flexibility in planning the walks. Furthermore, the patients will be encouraged to think about the opportunities for taking these extra steps and develop their own action plans. If patients have difficulties devising the action plan, the counsellor can suggest potential opportunities for including walking into their daily routine (walking as part of daily commuting, walking after a meal, walking with (grand)children, walking a dog, walking meetings, etc.).

The information collected during the first call will then be used by the counsellor to set and tailor the mHealth intervention. Specifically, the weekly goal will be used to inform the review of the weekly step goal and feedback on weekly performance, and the action plan will be used to set the content and timing of the respective reminders.

During the next calls (months 1 to 5), the counsellor will support the patients in reviewing their step goals and action plans, address the barriers to achieving their plans, advise them to seek social support, provide them with feedback, and encourage them to increase their walking pace and interrupt their prolonged sitting episodes regularly. During these calls, the counsellor can adjust the mHealth intervention and adapt it to changing patients’ needs. Specifically, if the patients regularly achieve their step goal, the counsellor can challenge them to increase it. The call at five months will be scheduled at least two weeks before the six-month assessment to avoid potential patients’ reactivity that could inflate their PA outcomes at six months.

After the assessment at six months, the counsellor will have a final contact with the patients to encourage them to maintain or further increase their achieved levels of PA and remind them that they will continue receiving text messages for six more months.

### Outcome measures

Outcomes will be assessed at baseline and 3, 6, and 12 months after randomisation, as described in Table 1. The primary outcome is defined a priori as the change in average daily step count from baseline to 12 months. All other outcomes and comparisons are defined as secondary outcomes.

Average daily step count and other measures of physical behaviour and sleep will be objectively assessed by accelerometry using Actigraph wGT3X-BT worn on a non-dominant wrist for seven days, 24 h a day. The measures will be derived from raw-accelerometry files using an open-access package GGIR in R according to standard procedures, including detection of non-wear and calculation of the average magnitude of dynamic acceleration corrected for gravity (Euclidean Norm minus 1 g) over 5-s epochs [76, 77]. Non-wear will be imputed using the default setting, and patients will be excluded if their post-calibration error is > 0.01 g (10 mg) or they have < 3 days of valid wear (defined as > 16 h per day), or their wear data are not present for each 15-min period of the 24 h cycle [78]. The other physical behaviour measures will include average acceleration, intensity gradient, and acceleration above which a person’s most active 10, 30, 120 and 480 min are accumulated, time spent sedentary and in sedentary bouts > 30 min, and time spent in light, moderate and vigorous PA [79, 80]. The sleep measures will be calculated using automated sleep detection [81], without using a sleep log, and will include sleep time (accumulated nocturnal sustained inactivity bouts), sleep window (time difference between falling asleep and waking up, including time being awake during the night), sleep onset and wake time, and sleep efficiency (ratio of sleep time compared with sleep window) [82].

Lower body strength will be assessed by a 30 s chair-stand functional test. The test is administered using a chair without arms, placed against a wall [83]. Fasting plasma glucose, glycated haemoglobin (HbA_1c_), and lipid profile will be assessed by standard laboratory or point-of-care methods available to GPs. The available methods may vary among GPs, but the GPs will be required to use the same method across all assessment points. Blood pressure, height, weight, and waist circumference will be measured to the nearest 1 mmHg, 1 cm, 1 kg, and 1 cm, respectively, using standard calibrated devices available in the GP's office. Symptoms of anxiety and depression will be assessed by the Hospital Anxiety and Depression Scale (HADS), a 14-item questionnaire consisting of depression and anxiety subscales [84]. Health-related quality of life will be assessed by the 12-Item Short Form Health Survey (SF-12) [85].

Patients' sociodemographic characteristics, education level, medical history, current smoking status, alcohol intake, and current medications will be collected (Table 1) and used as confounders in the analyses. In addition, the Morningness-Eveningness Questionnaire (MEQ) for the assessment of patients' chronotype [86] and a short version of the European Health Literacy Survey (HLS-Q12) [87] will be administered at baseline.

In addition to pre-/post-intervention outcomes, the Fitbit data will be used to explore short-term changes in behaviour proximal to the triggered text messages using a micro-randomisation design [88]. Briefly, HealthReact enables setting a probability that a triggered text message is actually sent out, thus enabling micro-randomisation. We will set the probability of sending out each triggered text message to 50% (i.e., only every other text message that is triggered will be actually sent out). Thus, we will be able to compare physical (in)activity, as recorded by Fitbit after the trigger, between text messages that were actually sent and those that were not. For different types of text messages, we will evaluate various time periods after the trigger: for just-in-time prompts to increase walking pace or interrupt sitting, we will evaluate periods ranging from 5 to 15 min; for the action plan reminders, we will evaluate periods ranging from one hour to one day; for the review of the weekly goal and the feedback on weekly performance, we will evaluate periods ranging from one day to one week. In addition, we will explore the moderating effect of the context (e.g., time of the day, day of the week, weather, actual patient performance) on the impact of individual text messages.

#### Adverse events

Adverse events will be monitored and recorded throughout the study period. Data regarding falls, injuries, musculoskeletal problems, hypoglycaemic episodes, major cardiovascular disease events, and any other events potentially related to the study implementation will be collected at all assessments.

### Data management and analysis

#### Data management

All study data will be collected and managed using secure REDCap electronic data capture tools hosted at Charles University, Prague [69]. To further ensure data confidentiality, data access rights to REDCap will be granted to only a few members of the research team and selectively constrained according to their roles in the trial. Patients' Fitbit data will be stored in a pseudonymised form on a secure HealthReact server. Patients' study information will not be released outside of the study without the patient's written permission.

The trial is not overseen by an independent data monitoring committee as it neither recruits vulnerable populations, poses any unusual risk to trial participants, nor assesses mortality or major morbidity outcomes [89, 90]. The trial results will be disseminated in open-access scientific journals regardless of the magnitude or direction of effect, and a completely deidentified dataset will be made openly available in an appropriate data repository.

#### Statistical analysis

The primary outcome (change in daily step count at six months) will be analysed using a linear mixed-effect model accounting for clustering at the GP level (random effect) and adjusted for baseline value, prediabetes/diabetes condition, and sex (fixed effects) [91]. The intervention effect (adjusted value for change in intervention minus change in control) will be reported as the mean (95% CI) and associated *p*-value (considered significant at p < 0.05). The intervention effect for secondary outcomes (Table 1) will be assessed using the same approach and reported as a mean (95% CI). However, individual *p*-values will not be reported for secondary outcomes as the adjustment for multiple testing will not be undertaken, and outcomes will be interpreted with caution in relation to the overall pattern of results. Assumptions of normality will be tested, and an alternative distribution will be used where necessary. Baseline characteristics will be compared between the intervention and control arms. If meaningful differences are demonstrated, the measure will be added to statistical models as a covariate. The analyses of the outcomes will be undertaken using both the intention-to-treat principle, considering all patients as randomised regardless of protocol adherence, and a per-protocol analysis, including only patients (a) syncing their Fitbit trackers at least once a month (both arms), (b) engaging in at least five phone counselling sessions, and (c) receiving text messages for the entire study duration (intervention arm only). The impact of missing data will be assessed using a sensitivity analysis, and missing data will be imputed using multiple imputations created by predictive models based on the patients with complete data [92]. All statistical analyses will be performed using an R environment for statistical computing.

### Process evaluation

The process evaluation will be conducted alongside the effectiveness evaluation to understand how the intervention is delivered and received and how this may affect the variation in outcomes. The framework for the process evaluation will be guided by the Medical Research Council (MRC) guidance for process evaluations of complex interventions [93] and will include both qualitative and quantitative components. In line with the MRC guidelines, we will investigate intervention aspects such as implementation (which comprises implementation process, reach, fidelity, dose and adaptations), mechanisms of impact, context, and the relations between these. We will use various data sources (forms filled by the GPs, notes taken by the counsellors, data from REDCap electronic data capture tools, data from Fitbit and HealthReact) to devise measures such as the percentage of patients who declined participation and reasons for their non-participation, number and duration of the phone counselling sessions, frequency of syncing the Fitbit monitor, number of just-in-time prompts, whether a patient received full or adapted mHealth intervention, etc. Selected measures will be used as predictor variables in linear models to estimate their effect on PA outcomes. In addition, we will conduct qualitative semi-structured interviews to explore the barriers and facilitators to the acceptability of and adherence to the intervention and to identify the factors that supported the improvement in physical behaviours of the patients from the intervention arm (Additional file 4).

#### Ecological momentary assessment study

As part of the process evaluation, we will conduct an Ecological Momentary Assessment (EMA) study [94] exploring patients’ perceptions of just-in-time text messages. In addition to triggering text messages, the HealthReact system comprises a mobile app that can display time- and event-based questionnaires. After two months of participating in the study, patients from the intervention arm who regularly sync their Fitbits (a proxy for continuous internet connection) will be instructed by the phone counsellor to install the HealthReact app on their smartphones and answer as many questionnaires as they possibly can for the rest of the 12-month study. The questionnaires will be delivered with a 50% chance 15 min after each just-in-time text message, but not more frequently than every other day, and expire after 10 min. The questionnaires will be based on the capability-opportunity-motivation-behaviours (COM-B) model. For example, to explore patients' capabilities, the questionnaires will include items on momentary fatigue and stress [95, 96]. To explore opportunities for behaviour change (i.e., increase the walking pace or interrupt sitting), the questionnaires will include questions on contextual barriers, such as walking in a company (which makes it impossible or socially unacceptable to increase pace) or sitting in a cinema (which makes it impossible to interrupt sitting). In addition, patients will be asked whether they were aware of the text message at all and, if so, whether they perceived it as being sent just in time, i.e. when they were actually walking or sitting.

## Discussion

The ENERGISED trial is a pragmatic randomised controlled trial that aims to assess the effectiveness of a mHealth intervention with lead-in phone counselling support that can be potentially translated to routine primary care. The trial has several important strengths.

Firstly, the control arm, against which the intervention will be compared, was designed as an active control. Patients allocated to the active control arm will receive from their GPs brief advice, including a recommendation to self-monitor their daily steps and try to achieve a specific step goal and a Fitbit tracker. The active control was primarily chosen to be able to isolate the net effect of the mHealth intervention. Indeed, given that self-monitoring using a simple activity tracker is effective in increasing PA levels, complex interventions should be compared against a control consisting of self-monitoring and a set goal to demonstrate their additional benefits above and beyond self-monitoring alone [38]. In addition, given the proven benefits of PA for diabetes prevention and management, we consider active control to be a more ethical choice than passive control. Besides, the use of active control has several other advantages: (A) It reflects the growing number of people already using some activity tracker for self-monitoring, thus, enabling their inclusion in the study and, consequently, being more ecologically valid. (B) It increases perceived value for patients even if they are allocated to the control group, thus, increasing the willingness of the patients to be enrolled in the study and limiting the self-selection bias. (C) Along the same lines, it also increases perceived convenience for GPs, making their recruitment for the study easier. (D) As both control and intervention arms receive from their GPs the same treatment, the GPs can be effectively blinded to the patient's allocation. Taken together, the choice of active control increases both the internal and external validity of the trial and is in line with its pragmatic design.

Secondly, also in line with the pragmatic trial design, we attempted to make the eligibility criteria as wide as possible. Thus, even though achieving the full potential of the intervention requires a smartphone with a mobile data plan, we allow for the inclusion of patients that only have a basic cell phone able to receive calls and text messages. These patients will receive an adapted version of the mHealth intervention, lacking the just-in-time messages, but equalised in terms of the number and types of messages. Consequently, the trial will enable the recruitment of older and lower socioeconomic status patients, thus increasing its external validity.

Thirdly, the recruitment process is designed to minimise previously described self-selection and selection biases [29, 63, 97, 98]. The self-selection bias commonly occurs when potentially eligible patients identified in GPs databases are recruited systematically, e.g., via mail invites. Usually, only a small fraction of patients answer these invites, and these patients are not representative of the target population: they are typically younger, more likely to be female, better educated and more physically active [97–99]. The self-selection bias can be reduced using opportunistic recruitment when GPs address patients coming to regular visits and invite them personally to participate [64]. However, this approach leads to selection bias, when GPs preferentially pick patients they believe benefit most from the intervention [29, 63]. In the ENERGISED trial, we apply opportunistic recruitment but try to minimise the selection bias by implementing the following procedures: (A) For each practice, we first compile a list of all potentially eligible patients and randomly select a limited sample available for recruitment. (B) We then ask GPs to address all patients from this sample, evaluate their eligibility, invite those who are eligible, and record eventual reasons for exclusion or patients' refusal to participate. (C) Only after a GP addresses all patients from the sample, we provide them with a new random sample until the recruitment is finished. The process is described in more detail in the Methods section. While this approach cannot completely eliminate the self-selection and selection biases, it will enable us to explore the potential extent and direction of the bias by providing a clear denominator and evaluate the feasibility of the intervention for translation into routine care.

Fourthly, the patients will be recruited from a relatively large number of 21 practices. This decision was primarily driven by the fact that the practices in the Czech Republic are relatively small, so a large number of practices is needed to recruit the required number of patients. Nevertheless, the large number of practices representing various urban and rural locations and geographic regions is also in line with the pragmatic design of the trial as it increases its external validity. Previous PA interventions in primary care that were initially shown to be effective in trials recruiting from a limited number of primary care practices failed to demonstrate benefits when translated to routine care [18–21]. Thus, including a large number of practices in the trial suggests that, if shown to be effective, the future translation of the intervention into routine care will be more likely to succeed.

Fifthly, while the main intervention goal (and the trial's primary outcome) is the increase in the daily number of steps, patients will be encouraged to pursue other goals: increase their walking pace and limit their prolonged episodes of sitting. All these goals align with the recent recommendations [10, 11], and a body of evidence suggests that increased walking pace and limited time spent in prolonged sitting bouts have beneficial effects for patients with type 2 diabetes, independent of the increases in the total daily step count [67, 100–102]. In addition, giving patients alternative goals can increase the chances that they will be able to pursue at least some of them and strengthen their sense of 'goal ownership' important for their motivation [74, 75]. The 'goal ownership' is further supported by negotiating their individual daily step goal during initial phone counselling sessions.

Sixthly, the mHealth intervention incorporates the latest technological advances to deliver text messages just in time and adapted to individual patients. The JITAIs have been recently shown to be effective in PA interventions in various populations [52, 53]; however, their effect in (pre)diabetes patients has not yet been explored. Future iterations of this intervention can implement advanced reinforcement learning algorithms to further improve their impact [103, 104].

Finally, the intervention is designed to be scalable so that it can be easily rolled out to routine use in primary care without prohibitive costs and additional burdens on the GPs. The costs include the initial provision of the Fitbit device, but it can be eventually replaced by a cheaper device or a free step-counting app [43]. The costs further include phone counselling during the lead-in phase, which is relatively resource-intensive but necessary to ensure proper implementation and tailoring of the mHealth component. To limit the costs of phone counselling, we recruited counsellors from among the university students and provided them with the necessary training. Following the lead-in phase, the mHealth intervention can be maintained in the long term without substantial costs that only include running a server and sending out text messages (which can be eventually replaced by free app notifications or WhatsApp messages). Importantly, the phone counselling provided by trained counsellors can compensate for the lack of expertise in PA counselling among the GPs. Thus, the intervention does not require either specific training of GPs or their extra time, which are significant barriers to providing PA interventions in primary care [28]. In summary, if shown to be effective, the intervention has the potential to be translated into routine primary care without obstacles on the GPs’ side.

The trial also has several limitations: (1) As GPs in the Czech Republic only follow prediabetes and uncomplicated type 2 diabetes patients not taking insulin, the findings of the trial cannot be generalised to insulin-dependent diabetes patients or those with high HbA_1c_ levels. (2) Despite our effort to keep GPs blinded to patient allocation, we cannot ensure it, as the allocation may become apparent during the discussions with patients at repeated visits. However, this will not impact outcome assessment. (3) The use of innovative technology can be a limitation for some patients who might struggle to set their Fitbits properly. However, this limitation is partly overcome by the provision of phone-based technical support. (4) The intervention only focuses on walking. Even though walking is the most common mode of PA in (pre)diabetes patients and is suitable for nearly everyone [105], it can still be a limitation for some patients who prefer to accumulate their PA by other means (e.g., cycling, swimming).

## Conclusions

There is an urgent need for effective, sustainable, and scalable PA interventions for (pre)diabetes patients that can be translated to routine primary care [17, 22]. Based on recent evidence and harnessing the latest technology advances, we have developed an mHealth intervention based on JITAI principles to be introduced to the patients by their GPs with initial lead-in support by trained phone counsellors. The intervention will be tested in the pragmatically designed randomised controlled trial ENERGISED, with the primary outcome being a change in daily step count assessed at 12 months. In addition, we will conduct an extensive process evaluation using mixed methods and Ecological Momentary Assessment to be able to further improve the next iteration of the intervention. If shown to be effective, we will seek to implement the intervention as part of the standard primary care in the Czech Republic for (pre)diabetes patients and potentially other patient populations, potentially leading to important public health benefits.

## Supplementary Information

**Additional file 1.** Standard Protocol Items: Recommendations for Interventional Trials (SPIRIT) 2013 Checklist.

**Additional file 2.** Template for Intervention Description and Replication (TIDieR) Checklist.

**Additional file 3.** PRagmatic Explanatory Continuum Indicator Summary (PRECIS-2) table of scores for trial domains and the PRECIS-2 wheel scheme.

**Additional file 4.** Protocol for the qualitative study conducted as part of the process evaluation of the ENERGISED trial.

## Data Availability

Not applicable.
