# Supplementary material for: mHealth intervention delivered in general practice to increase physical activity and reduce sedentary behaviour of patients with prediabetes and type 2 diabetes (ENERGISED): rationale and study protocol for a pragmatic randomised controlled trial
Source: BMC Public Health. 2023 Mar 31;23:613. doi: 10.1186/s12889-023-15513-1 (PMC10064755; doi:10.1186/s12889-023-15513-1)

**Additional file 3.** **PRagmatic Explanatory Continuum Indicator Summary (PRECIS-2) table of scores for trial domains and the PRECIS-2 wheel scheme**

|  | Domain | Score | Rationale |
| --- | --- | --- | --- |
| 1 | Eligibility - Who is selected to participate in the trial? | 4 | All patients followed for type 2 diabetes and prediabetes at the general practice are eligible if they are regular users of a mobile phone (not necessarily a smartphone). Exclusion criteria include patients that are: (a) pregnant, (b) having a household member already recruited for the trial, (c) living in a residential or nursing home, (d) having co-morbidities such as active malignancy, recent myocardial infarction, a renal disease requiring dialysis, etc. |
| 2 | Recruitment - How are participants recruited into the trial? | 5 | Only patients appearing in the general practice for a routine health check-up are recruited. |
| 3 | Setting - Where is the trial being done? | 5 | Twenty-one general practices representing various urban and rural locations and geographic regions across the country. |
| 4 | Organisation - What expertise and resources are needed to deliver the intervention? | 5 | The participating general practitioners were trained remotely in three separate sessions (each lasting approximately one hour); however, only a minor part of these sessions was dedicated to the provision of the intervention. These training sessions are considered part of the intervention. |
| 5 | Flexibility - How should the intervention be delivered? | 5 | Following the training, the details of how to implement the intervention are left up to the general practitioners. |
| 6 | Flexibility - What measures are in place to make sure participants adhere to the intervention? | 5 | Patients are encouraged to adhere to the intervention by phone counsellors; however, this is considered part of the intervention. |
| 7 | Follow-up - How closely are participants followed-up? | 4 | For patients with type 2 diabetes, all assessments were part of their regular check-up visits. For patients with prediabetes, the assessment at 3 months required an additional visit to their general practitioner. If patients did not show up for regular check-up visits, the doctors were reminded to contact them. |
| 8 | Primary outcome - How relevant is it to participants? | 4 | The daily number of steps is highly relevant to the patients and their general practitioners and can be easily assessed using consumer-level activity trackers. However, for the purpose of the trial, we assessed the daily number of steps using a research-grade accelerometer that is not commonly used in general practices. |
| 9 | Primary analysis - To what extent are all data included? | 5 | Intention-to-treat analysis using all available data. |


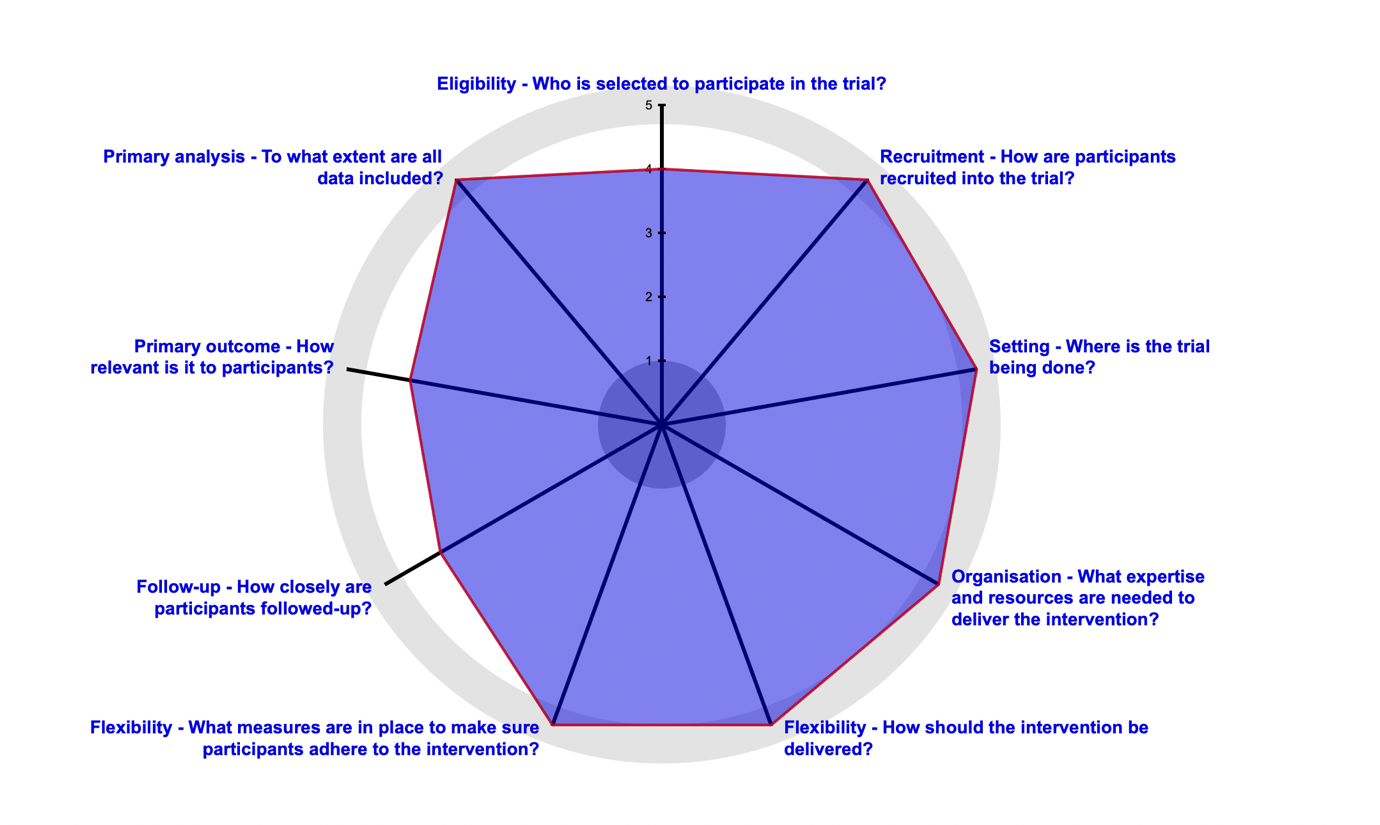

Supplement: Supplementary file 3 — Additional file 3. PRagmatic Explanatory Continuum Indicator Summary (PRECIS-2) table of scores for trial domains and the PRECIS-2 wheel scheme. [file 12889_2023_15513_MOESM3_ESM.docx]
