# Supplementary material for: mHealth intervention delivered in general practice to increase physical activity and reduce sedentary behaviour of patients with prediabetes and type 2 diabetes (ENERGISED): rationale and study protocol for a pragmatic randomised controlled trial
Source: BMC Public Health. 2023 Mar 31;23:613. doi: 10.1186/s12889-023-15513-1 (PMC10064755; doi:10.1186/s12889-023-15513-1)
Supplement: Supplementary file 4 — Additional file 4. Protocol for the qualitative study conducted as part of the process evaluation of the ENERGISED trial. [file 12889_2023_15513_MOESM4_ESM.docx]

**Additional file 4.** **Protocol for the qualitative study conducted as part of the process evaluation of the ENERGISED trial**

The qualitative interview study aims to explore the barriers and facilitators to the acceptability of and adherence to the intervention and to identify the factors that supported the improvement in physical behaviours of the patients from the intervention arm. As part of the consent process for the trial, we will ask all patients for their permission to contact them and invite them to participate in an interview about their experience of the trial. We will purposively select 20 to 30 patients with high and low levels of adherence to the intervention and those who did and did not increase their daily step count. To ensure we capture patients' recent experiences of receiving the intervention and to avoid the interviews affecting trial outcomes, patients will be approached after the assessment at six months. Interviews will be conducted by phone. At the start of the interview, verbal consent will be recorded digitally. Each patient being interviewed will be offered a 20-EUR voucher to compensate them for their time.

Interview questions will explore the motivation for study participation, experiences of and views on the intervention and reasons for high or low adherence. The topic guide for the interviews will be informed by the Theoretical Domains Framework (TDF) to ensure broad coverage of factors influencing acceptability and feasibility, and we will use the capability-opportunity-motivation-behaviours (COM-B) model to prompt individuals to discuss barriers and facilitators to adherence relating to issues of capability, opportunity, and motivation. To ensure a comprehensive set of questions that address acceptability, we will also cover acceptability issues identified in the theoretical framework of acceptability (TFA), i.e., affective attitude, burden, perceived effectiveness, ethicality, intervention coherence, opportunity costs, and self-efficacy. The topic guide will be reviewed by Patient and Public Involvement (PPI) representatives.

Semi-structured interviews, conducted by a university researcher or phone counsellors involved in the trial, will last up to 45 minutes and be digitally recorded and then transcribed verbatim by an independent transcriber. The verbatim transcripts of the interviews will be analysed using thematic analysis. Thematic analysis will be both inductive, guided by patients' accounts, and deductive, informed by TDF and COM-B. Using NVivo, two experienced qualitative researchers will independently code the transcripts. An iterative approach will be adopted whereby coded themes will be used as the categories for analysis, which will be refined and discussed with the rest of the research team until a consensus is reached. To indicate the frequency with which themes are provided by patients, we will use the terms “all”, “almost all”, “most”, “the majority”, “some”, and “a few”. All transcripts will be coded using this approach.
